# Phosphorus’s Ameliorative Effect on High Level Bacterial Protein-Induced Metabolic Disorders: Alleviating Oxidative Stress and Lipid Dysregulation in *Procambarus clarkii*

**DOI:** 10.3390/antiox15010028

**Published:** 2025-12-24

**Authors:** Jiarong Guo, Linlin Yang, Dongwu Wang, Minglang Cai, Jinlong Li, Xin Tian, Xiudan Yuan, Yi Hu, Zhigang He

**Affiliations:** 1 Hunan Fisheries Science Institute, No. 728 Shuanghe Road, Changsha 410153, China; guojr9913@hunaas.cn (J.G.); wangdw@hunaas.cn (D.W.); jinlong-l@hunaas.cn (J.L.); tianx2323@hunaas.cn (X.T.); xiudanyuan@hunaas.cn (X.Y.); 2 Yuelushan Laboratory, Changsha 410128, China; 3 Fishery College, Hunan Agricultural University, Changsha 410128, China

**Keywords:** *Procambarus clarkii*, phosphorus, *Clostridium autoethanogenum* protein, lipid metabolism, oxidative damage

## Abstract

A 10-week growth experiment was conducted to evaluate the physiological effects of dietary phosphorus supplementation on red swamp crayfish (*Procambarus clarkii*) feeding diets with high *Clostridium autoethanogenum* protein (CAP) levels. Six isonitrogenous and isolipid diets were formulated: The FM diet contained 10% fishmeal, which is equivalent to a dietary phosphorus level of 1.41%, and the CAP, CAPSP1, CAPSP2, and CAPSP3 diets substituted all fishmeal with CAP and supplemented with 0, 2.5%, 3%, and 3.5% Ca(H_2_PO_4_)_2_, respectively (corresponding to dietary phosphorus levels of 0.66%, 1.27%, 1.40%, and 1.52%). A total of 600 crayfish with an initial mean weight of (5.01 ± 0.02) g were selected and randomly assigned to 15 cages for feeding and sampled at the end of the experiment. Results indicate that high-dose CAP replacing fishmeal caused abnormal hepatopancreatic tissue structure in crayfish, exacerbating lipid deposition and oxidative stress. Compared with the CAP group, the specific growth rate (SGR) of crayfish in the CAPSP2 and CAPSP3 groups significantly increased (*p* < 0.05). The activities of antioxidant enzymes and lipid-degrading enzymes in the hepatopancreas, along with the relative expression of related genes, were significantly enhanced (*p* < 0.05). Metabolomic analysis demonstrated significant differences in major differential metabolites and metabolic pathways between the CAP group crayfish and the CAPSP2 group (*p* < 0.05). CAPSP2 group crayfish exhibited a higher content of phosphatidylcholine (PC) and lysophosphatidylcholine (LPC), with significant enrichment in glycerophospholipid metabolism and fatty acid metabolism pathways (*p* < 0.05). Overall, supplementing dietary phosphorus levels to 1.40–1.52% effectively mitigated growth retardation, oxidative damage, and lipid metabolism disorders induced by high-proportion CAP replacement of fishmeal.

## 1. Introduction

Aquaculture is one of the fastest-growing food sectors worldwide, and it has progressively become the primary method for increasing global aquatic product supply over the past two decades, making significant contributions to providing high-quality aquatic animal foods for humanity [1,2]. The future growth of this industry requires both sufficient aquatic feed production to meet market demand and higher standards for protein sources [3]. Fishmeal, as the most common high-quality protein source, offers advantages such as high protein content, balanced nutrients, and excellent palatability [4]. However, the scarcity of fishmeal resources and its continuous price increases have become bottlenecks for aquaculture development. Therefore, developing high-quality alternative protein sources to fishmeal has become an urgent issue for the healthy development of the aquaculture industry.

*Clostridium autocthanogenum* protein (CAP), a by-product derived from Clostridium autoethanogenum, is a novel single-cell microbial protein obtained through chemical reactions involving CO and ammonia water reacting with H_2_, followed by anaerobic fermentation and precipitation purification processes [5]. It contains over 80% crude protein and is virtually free of antinutritional factors and *Salmonella*. CAP offers advantages such as high production efficiency, no requirement for arable land, and environmental sustainability. Furthermore, CAP exhibits amino acid and fatty acid compositions similar to fishmeal, positioning it as a strong alternative [6,7]. Its feasibility as a fishmeal substitute in aquaculture feeds has been validated across multiple studies. For instance, studies on Jian carp (*Cyprinus carpio* var. Jian) [8], largemouth bass (*Micropterus salmoides*) [9] and grass carp (*Ctenopharyngodon idella*) [10] demonstrated that replacing up to 60% of fishmeal with CAP in feed did not impair growth performance. Research on tilapia (*Oreochromis niloticus*) [6], black seabream (*Acanthopagrus schlegelii*) [11] and largemouth bass [12] found that the addition of appropriate amounts of CAP to feed improved the growth performance, muscle quality, and serum biochemical parameters in cultured animals. However, replacing over 50% of fishmeal with CAP in Pacific white shrimp (*Litopenaeus vannamei*) feed significantly reduced growth performance while markedly decreasing free amino acid and flavor amino acid content in shrimp muscle [13]. Huang noted that replacing over 60% of fishmeal with CAP caused liver damage and reduced antioxidant activity in pearl gentian grouper (*Epinephelus fuscoguttatus*) [14]. Furthermore, when fishmeal replacement exceeded 60%, turbot exhibited exacerbated lipid deposition and lipid metabolism disorders [15]. Thus, excessively high CAP replacement of fishmeal can produce adverse effects, highlighting the need to identify and overcome the limiting factors behind these negative outcomes.

CAP constitutes an excellent replacement for fishmeal because of its high nutritional content and safe, eco-friendly qualities [16]. However, as shown in Table S1, compared to fishmeal, CAP exhibited significantly lower levels of phosphorus, fat, and certain amino acids, which could potentially limit its use as a high-ratio replacement for fishmeal [17]. As an essential nutrient for crustaceans, phosphorus serves as a key synthetic substrate for skeletal tissue and plays vital roles in energy metabolism, biomolecular synthesis, and intracellular signaling pathways [18,19]. Phosphorus deficiency reduces growth performance and digestive enzyme activity in aquatic organisms, leading to abnormal lipid deposition, oxidative stress, and disrupted lipid metabolism [20,21]. Due to crustaceans’ relatively low efficiency in absorbing phosphorus from water bodies, the primary source of phosphorus required by their bodies is feed [22,23]. Studies on rainbow trout (*Oncorhynchus mykiss*) [24] and Japanese flounder (*Paralichthys olivaceus*) [25] indicated that adding appropriate amounts of phosphorus to feed promoted fat metabolism in fish and reduced fat deposition. Furthermore, Zheng et al. [17] indicated that supplementing phosphorus in feed for Pacific white shrimp alleviated oxidative stress and intestinal tissue damage induced by CAP, suggesting phosphorus could be a key limiting factor when replacing fishmeal at high ratios. However, few studies have explored the potential for high levels of fishmeal substitution by CAP from the perspective of phosphorus requirements.

Most phosphorus in aquatic animals is stored in mineralized tissues as hydroxyapatite [Ca_10_(PO_4_)_6_(OH)_2_], while a portion functions biologically as phospholipids [26]. Among these, glycerophospholipids constitute the most abundant phospholipid class in living organisms. Beyond forming biological membranes, they act as membrane surfactants involved in physiological activities such as protein recognition and cell membrane signal transduction [27,28]. During glycerophospholipid synthesis and metabolism, phospholipids undergo hydrolysis by phospholipases to yield lysophosphatidylcholine (LPC) and lysophosphatidic acid (LPA) [29]. LPC catalyzes the conversion of dipalmitoylphosphatidylcholine to phosphatidylcholine (PC) [30]. As the most abundant glycerophospholipid, PC can be hydrolyzed by phospholipase C or D into diacylglycerol (DAG), thereby regulating glycerophospholipid metabolism by enhancing enzyme activity in processes such as phosphatidylethanolamine (PE) synthesis [31]. Although one study demonstrated that phosphorus deficiency significantly reduces PC and PE levels, leading to lipid metabolism disorders [32], current research on phosphorus in aquatic animals is generally limited to the domains of demand and energy metabolism. Studies exploring the association between phosphorus and phospholipid metabolism remain relatively scarce.

As the fourth most cultivated species in China’s freshwater aquaculture, the red swamp crayfish (*Procambarus clarkii*) has the advantages of rapid growth, high protein content, and strong stress resistance, with a production volume reaching 3.447 million tons in 2024 [33]. With the continuous expansion of farming areas and production volumes, the healthy growth and stable returns of crayfish require a nutritionally balanced and cost-effective compound feed. Developing and applying CAP as an alternative protein source could simultaneously address nutritional needs and environmental protection. In our previous research, completely replacing fishmeal with CAP inhibited crayfish growth performance and immunity while causing hepatopancreatic damage [34]. Given phosphorus’s critical role in aquatic animal growth and vital functions, it is reasonable to infer that these outcomes are closely linked to phosphorus levels in diets. Therefore, based on the aforementioned findings and referencing previously reported phosphorus requirements for crayfish [35], this study was therefore designed to systematically evaluate the effects of dietary phosphorus supplementation on high-CAP-diet-fed crayfish, with a focus on growth performance, hepatopancreatic antioxidant capacity, and lipid metabolism. To delve deeper into the mechanistic underpinnings, non-targeted metabolomics was further employed to explore how phosphorus modulates the metabolic utilization of CAP.

## 2. Materials and Methods

### 2.1. Ethic Statement

This study was approved by the HUNAU Animal Care and Use Committee (Granted number 2024EA126). All of the experimental procedures involving animals were conducted in accordance with the rule of Care and Use of Laboratory Animals in China, Animal Ethical and Welfare Committee of China Experimental Animal Society.

### 2.2. Diets and Feeding Experiment

**Figure 1:**
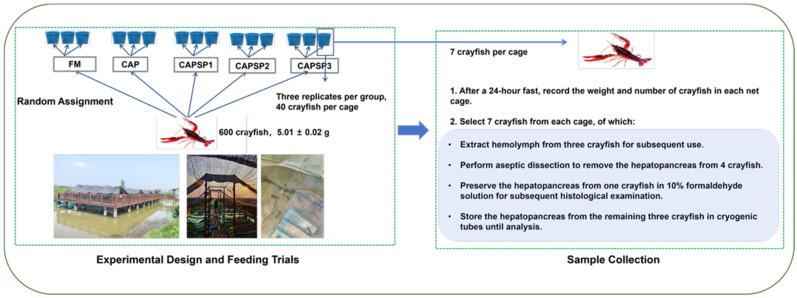
Experimental protocol and design.

In this study, five isonitrogenous and isofat diets (FM, CAP, CAPSP1, CAPSP2, CAPSP3) were formulated to feed crayfish (Figure 1). Based on the previous studies in our laboratory, a basal diet containing 10% fishmeal was used as a positive control diet (FM) and CAP was used to replace all fishmeal proteins in the diets and supplemented with fish oil until the lipid levels were consistent with those of the FM diets, which served as a negative control diet (CAP). The phosphorus content of each feed ingredient is shown in Table S2. Ca(H_2_PO_4_)_2_ was selected as the phosphorus source due to its widespread use, excellent solubility, and high utilization rate by crayfish. Since the phosphorus content of CAP was lower than that of fishmeal, the three other experimental groups were supplemented with 2.5% (CAPSP1), 3.0% (CAPSP2) and 3.5% (CAPSP3) of Ca(H_2_PO_4_)_2_·on top of the CAP group to investigate the effect of different phosphorus levels on crayfish feeding on high levels of CAP. The feed preparation procedure was based on earlier publications [36]. To prepare dough, the materials were first crushed, sieved through a 60-mesh sieve, and mixed. Separately, deionized water and blending oil were then added, and everything was well combined. Using a feed pelletizer, the feed was formed into pellets about 2 mm in size. It was then air-dried in a cool location, sealed in bags, and kept at −20 °C until it was needed. Table 1 displays the five trial diets’ formulas and approximate nutritional composition.

Crayfish were acquired from Guolian Food Co., Ltd.’s hatchery in Yiyang, Hunan, China, where the feeding trials were carried out. The design of the ten-week trial was as follows: two weeks of commercial feed before the commencement of the formal trial. At the conclusion of the acclimatization period, 15 (five groups of three replicates of 40 crayfish each) clean and disinfected net cages (2.0 m × 2.0 m × 1.5 m) were randomly assigned to 600 healthy, evenly sized crayfish. The treatment and cage location for each replicate were also randomly assigned. At the start of the trial, the crayfish weighed an average of 5.01 ± 0.02 g. Two times a day (7:30–8:30 and 17:00–18:00), the crayfish were fed ad libitum at a rate of 8% of their body weight. The feeding rate was changed every week based on the body. Throughout the feeding trial, the following water quality parameters were maintained: ammonia level ≤ 0.03 mg/L, pH 7.2–8.3, water temperature 26–27 °C, and dissolved oxygen > 6.0 mg/L.

### 2.3. Sample Collection

Following a 24-h fast, the crayfish were weighed and sampled at the conclusion of the feeding trial, and the weight and quantity of crayfish in each net cage were noted. Using a 1 mL sterile syringe, hemolymph was extracted from the cephalothorax of nine crayfish per group (three crayfish per net cage), maintained at 4 °C for the night, and then extracted by centrifugation (3500 rpm, 10 min, 4 °C) and kept at −80 °C for subsequent use. Four crayfish’s hepatopancreas were extracted out of each net box, and one crayfish’s hepatopancreas was preserved in a 10% formaldehyde solution for further histological examination; the remaining three crayfish were separated from their hepatopancreas, rinsed with PBS buffer, immediately submerged in liquid nitrogen, and then stored at −80 °C until analysis.

### 2.4. Analysis of Basic Composition of Diet

The nutritional levels of feed, including crude protein and crude fat, were determined according to methods described by the AOAC [37]. Specifically, crude protein was measured using the Kjeldahl method (AOAC Method 2001.11), and crude fat was determined via the Soxhlet extraction method (AOAC Method 920.39). Furthermore, an inductively coupled plasma optical emission spectrometer (Prodigy Plus, ICP-OES, Leeman, Milwaukee, WI, USA) was used to measure the phosphorus levels in feed in compliance with the reference standard (GB/T 6437-2018 [38]).

### 2.5. Analysis of Hemolymphatic and Hepatopancreatic Biochemical Indicators

Total antioxidant capacity (T-AOC), aspartate aminotransferase (AST), alanine aminotransferase (ALT), catalase (CAT), superoxide dismutase (SOD), glutathione peroxidase (GSH-PX), and the concentrations of malondialdehyde (MDA), triglycerides (TG), total cholesterol (TC), low density lipoprotein (LDL-C), and high density lipoprotein (HDL-C) were measured using commercial kits from Nanjing, China. Additionally, the ELISA kits (Nanjing JianCheng Bioengineering Institute, Nanjing, China) were used to measure the expression levels of FAS, ACC, and CPT-1 in the hepatopancreas. The manufacturer’s instructions were followed when conducting the experiments.

### 2.6. Real-Time Quantitative PCR

Total RNA was isolated from the hepatopancreas samples using the Total RNA Extraction Kit (Takara, Dalian, China). A Nucleic Acid Protein Analyzer (NanoDrop, 2000, Wilmington, DE, USA) was used to estimate the concentration and purity of total RNA. The Prime ScriptTM RT Kit (Takara, Dalian, China) was then used for immediate reverse transcription. After 1% agarose gel electrophoresis was used to assess the purity of the total RNA, the SYBR Prime Script RT-PCR Kit II (Takara, Dalian, China) was used to produce the reaction system. Real-time PCR was then performed on the CFX96TM Real-time PCR Detection System (Bio-Rad, Hercules, CA, USA). Table 2 offers a list of primer sequences and sources. The 2^−ΔΔCT^ method was used to normalize the levels of *sod*, *gpx*, *fas*, *srebp-1*, *acc2*, *acox*, *cpt-1* and *atgl* gene expression to *β*-actin [39].

### 2.7. Histological Morphological Analysis

Sections of hepatopancreatic tissue were taken in accordance with a prior study [40]. Hematoxylin and eosin (H&E) was used to stain sections (5 μm), which were then captured on camera using a microscope (Pannoramic SCAN II, 3DHISTECH KFT, Budapest, Hungary). In addition, a portion of hepatopancreas samples were sectioned in a cryosectioner, fixed in 4% paraformaldehyde for 10 min, and then stained with Oil Red O. The method for measuring the relative area of lipid droplets after Oil Red O staining was as follows: Select the same region for field of view examination. For each sample, randomly select and save six fields of view. Using the color region statistics function in ImageJ (v.1.8.0.112), the relative area of Oil Red O-stained lipid droplets was measured by calculating the proportion of red color in the entire field of view. Statistical analysis was then performed on the six data points. The selected fields of view must be random, and all areas, regardless of color intensity, were included in the statistics. The field selection method for measuring the relative area of vacuoles is similar to that for measuring the relative area of Oil Red O-stained lipid droplets. The primary focus was on calculating the relative area of vacuoles within the entire field of view, including all vacuoles regardless of size.

### 2.8. Analysis of Metabolomics

A UPLC system (Orbitrap Exploris 120, Thermo, Waltham, MA, USA) with an ACQUITY UPLC HSS T3 column (100 Å, 1.8 µm, 2.1 mm × 100 mm) was used to separate and identify hemolymph. Water with 0.1% formic acid (mobile phase A) and acetonitrile with 0.1% formic acid (mobile phase B) made up the mobile phase. The mass spectrometer, controlled by Xcalibur software (version 4.7, Thermo, MA, USA), performed DDA mass spectrometry data acquisition in both positive and negative ion modes. For peak extraction, alignment, and correction, we imported the raw data from the mass spectrometer into the commercial program Compound Discoverer 3.3 (version 3.3.2.31, Thermo, Waltham, MA, USA). Filter out peaks were not detected in over 50% of QC samples. We filled missing values for undetected peaks using the Fil Gaps algorithm within the software, then normalized the data using the Sum peak area. The self-built PSNGM Database.mzCloud online library (https://www.mzcloud.org/(accessed on 18 October 2024)), LIPID MAPS (https://www.lipidmaps.org/ (accessed on 18 October 2024)), HMDB (https://hmdb.ca(accessed on 18 October 2024)), MoNA (https://mona.fiehnlab.ucdavis.edu/ (accessed on 18 October 2024)), and NIST 2020 MSMS spectral library were used to identify the metabolites. The MS2 Match Factor Threshold was set at 50, and the MS1 mass tolerance was set at 5 ppm.

In the R project, ggplot2 (version 3.4.1) was used to create violin plots for metabolite abundances and expression abundance density plots. The Pheatmap package (version 1.0.12) in R was used to perform bidirectional clustering of both samples and metabolites while clustering analysis was applied to metabolite abundance values. A heatmap was drawn to visualize the abundance profiles of metabolic products in the samples. Using the R package Ropls (version 4.0.3) for dimensionality reduction, principal component analysis (PCA), partial least squares discriminant analysis (PLS-DA), and orthogonal partial least squares discriminant analysis (OPLS-DA) were applied to the sample data to reveal variations in metabolic composition between samples. The heatmap package (version 1.0.12) in R was employed to conduct cluster analysis on the abundance of differentially expressed metabolites and generate heatmaps. Venn diagrams for differentially expressed substances between the two groups were generated using VennDiagram (version 1.7.3). Correlation analysis of differential metabolites was performed with corrplot (version 4.0.3), and box plots were generated via ggplot2 (version 3.4.1) to display the distribution patterns of metabolite abundances across different groups. Further machine learning analysis (mlr3verse, version 0.2.7) was applied to the differentially expressed metabolites to extract key product information. Functional analysis of the differentially expressed metabolites primarily employed clusterProfiler (version 4.6.0) for KEGG enrichment analysis.

### 2.9. Statistical Analysis

SPSS 22.0 (IBM Corp., Armonk, NY, USA) was used for statistical analysis. Mean ± standard error was used to report quantitative data. Normality and homogeneity of variances were assessed via Shapiro–Wilk and Levene tests, respectively (Tables S3 and S4). To assess differences across groups, data were analyzed using a one-way ANOVA with the Tukey HSD multiple comparisons test; *p* < 0.05 indicated significant differences. Regression analysis also revealed both linear and quadratic trends (Table S5). To confirm the ANOVA results, we used the MANOVA method to highlight statistical differences between the evaluated groups (Table S6).

The statistical model for analysis of ANOVA is as follows:

*Y_ij_* = *μ* + *T_i_* + *e_ij_*

where *Y_ij_*, *μ*, *T_i_* and *e_ij_* denote the dependent variable observation, overall mean, fixed treatment effect and residual error, respectively.

## 3. Results

### 3.1. Effect of Phosphorus on Growth and Lipid Content of Crayfish Feeding on High Levels of CAP

**Figure 2:**
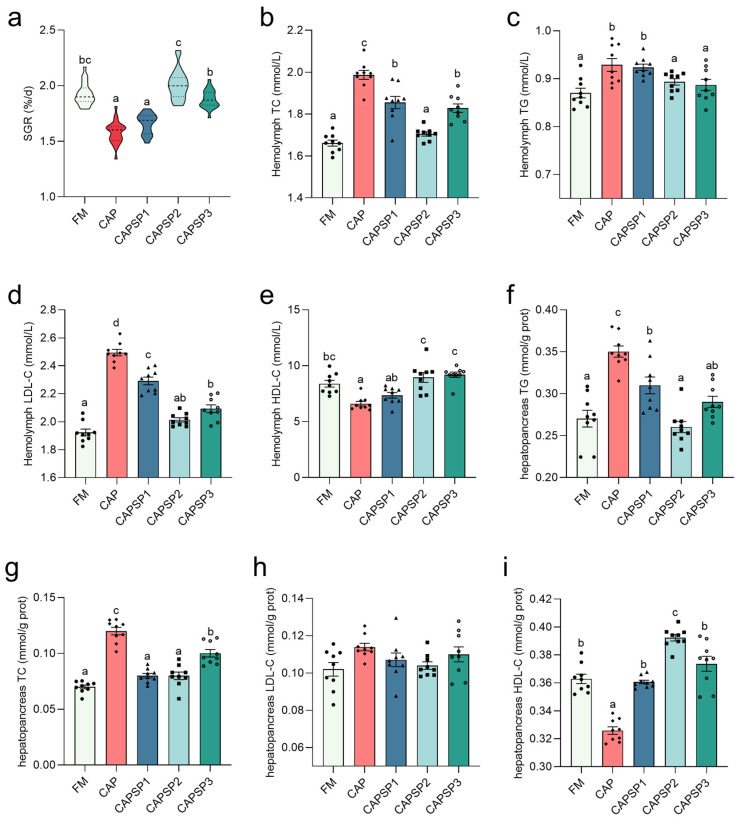
Effects of phosphorus on growth and lipid content in crayfish fed high levels of CAP. (**a**) Specific growth rate (SGR), SGR (%/d) = 100 × (ln final body weight − ln initial body weight)/days. (**b**–**i**) Contents of TG, TC, LDL-C and HDL-C in hemolymph and hepatopancreas. Different letters indicate significant differences between groups (*p* < 0.05).

The results showed that the SGR of crayfish in the FM, CAPSP2 and CAPSP3 groups were significantly (*p* < 0.05) higher than those in the CAP and CAPSP1 groups (Figure 2). Compared with the FM group, the CAP group exhibited significantly (*p* < 0.05) elevated contents of TG and TC in both hemolymph and hepatopancreas, alongside a marked reduction (*p* < 0.05) in HDL-C. When compared with the CAP group, all phosphorus-supplemented groups—particularly the CAPSP2 group—demonstrated a significant (*p* < 0.05) decrease in TG, TC, and LDL-C contents in both hemolymph and hepatopancreas, alongside a significant (*p* < 0.05) increase in HDL-C content. There was no significant (*p* > 0.05) difference in hepatopancreas LDL-C content among the groups.

### 3.2. Effect of Phosphorus on the Hepatopancreatic Histological Morphology of Crayfish Feeding on High Levels of CAP

**Figure 3:**
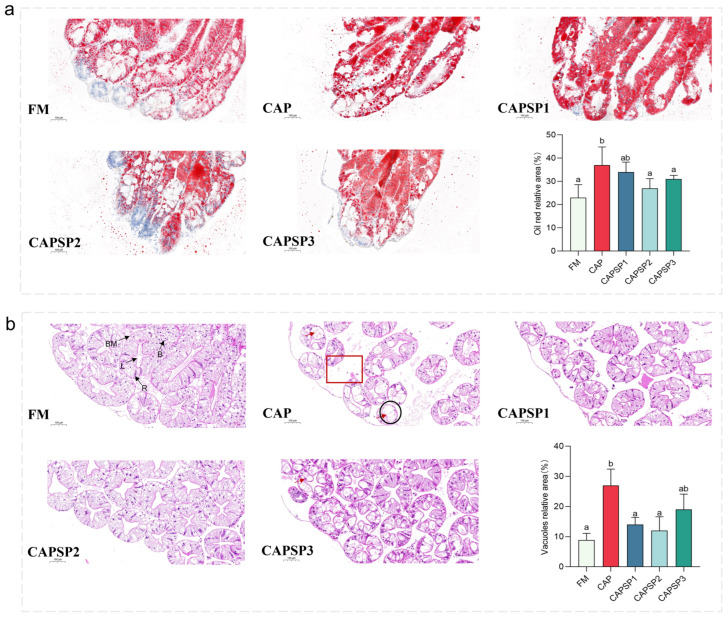
Effects of phosphorus on the histological morphology of hepatopancreas in high-level CAP-fed crayfish and statistical results. (**a**) Hepatopancreatic sections from each group were stained with Oil Red O and examined at 200× magnification in the same field of view at a uniform location. Subsequently, statistical analysis was performed based on the relative lipid droplet area measured in six randomly selected fields of view across all groups. (**b**) Hepatopancreatic sections from each group were examined at 200× magnification in the same field of view after hematoxylin and eosin (H&E) staining. Subsequently, statistical analysis was performed based on the relative vacuolar area measured in six randomly selected fields of view across all groups. B, B-cells; BM, basement membrane; R, R-cells; L, lumen of tubules. The red box and black circle indicate the hepatopancreatic tubular spaces, while the red arrow points to hepatopancreatic vacuoles. Different letters indicate significant differences between groups (*p* < 0.05).

Oil Red O staining of histological sections revealed the distribution of lipid droplets in the hepatopancreas across different crayfish groups (Figure 3a). Quantitative analysis indicated that the CAP group exhibited the largest relative area of lipid droplet distribution, significantly (*p* < 0.05) higher than the FM, CAPSP2, and CAPSP3 groups.

H&E staining revealed that compared to the FM group, crayfish in the CAP group exhibited abnormal hepatopancreatic structure, characterized by irregularly shaped hepatopancreatic tubule lumens, loosely arranged tubules with enlarged interstitial spaces, and conspicuous lipid droplets and vacuoles (Figure 3b). In contrast, the hepatopancreas of Procambarus clarkii in the CAPSP2 and CAPSP3 groups exhibited restored structural integrity, with densely arranged hepatocytes, continuous basement membranes, and hepatocytes displaying a star-shaped lumen with intact morphology. Quantitative analysis further indicated that the relative vacuolar area of the hepatopancreas in the CAP group crayfish was significantly (*p* < 0.05) higher than that in the FM group, CAPSP1 group, and CAPSP2 group.

### 3.3. Effect of Phosphorus on the Hepatopancreatic Function and Antioxidant Status of Crayfish Feeding on High Levels of CAP

**Figure 4:**
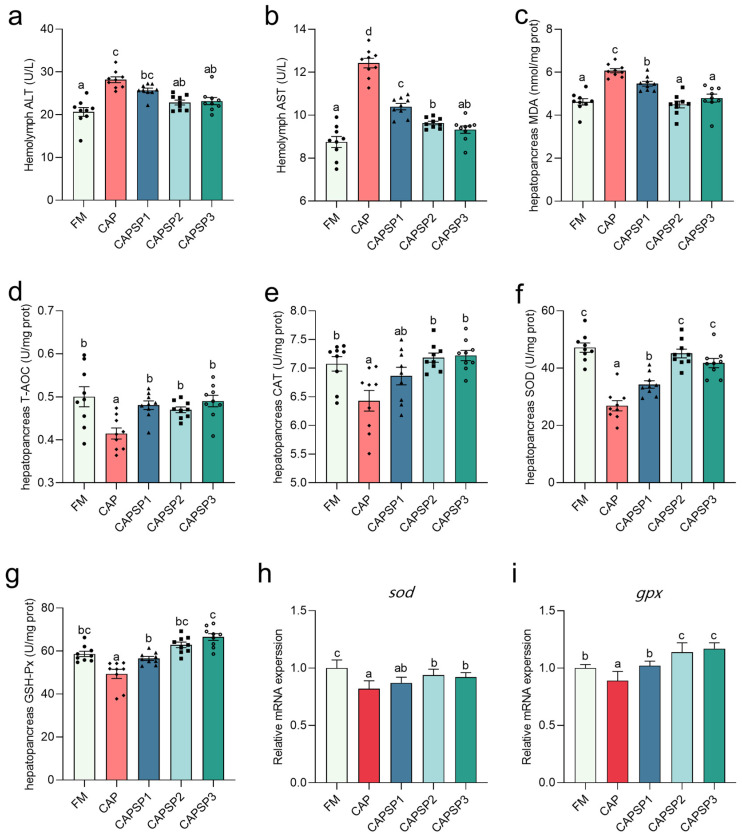
Effects of phosphorus on the hepatopancreatic function and antioxidant status of crayfish fed high levels of CAP. (**a**,**b**) Activities of ALT and AST in hemolymph. (**c**) Content of MDA in hepatopancreas. (**d**–**g**) T-AOC and activities of CAT, SOD and GSH-Px in hepatopancreas. (**h,i**) Relative gene expression of *sod* and *gpx* in hepatopancreas. Different letters indicate significant differences between groups (*p* < 0.05).

The results showed that the activities of AST and ALT activities in the hemolymph and content of hepatopancreatic MDA of crayfish in the CAP group was significantly (*p* < 0.05) higher than the rest of the groups, and the T-AOC was significantly (*p* < 0.05) lower than the rest of the groups (Figure 4). The activities of hepatopancreatic CAT, SOD, and GPX of crayfish in the FM, CAPSP2, and CAPSP3 groups were significantly (*p* < 0.05) higher than that of the CAP group. In addition, the relative gene expression of hepatopancreatic *sod* and *gpx* of crayfish in the CAP group was significantly (*p* < 0.05) lower than that in the FM, CAPSP2 and CAPSP3 groups.

### 3.4. Effect of Phosphorus on Indicators Related to Lipid Metabolism of Crayfish Feeding on High Levels of CAP

**Figure 5:**
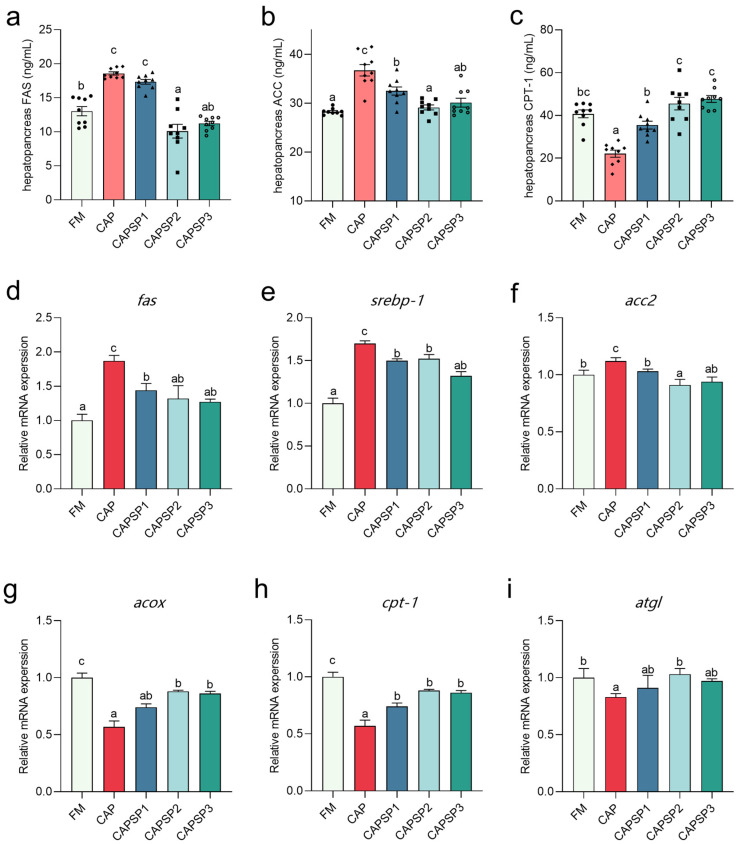
Effects of phosphorus on the activity of enzymes and relative gene expression related to lipid metabolism in the hepatopancreas of crayfish fed high levels of CAP. (**a**–**c**) Activities of FAS, ACC and CPT-1 in hepatopancreas. (**d**–**i**) Relative gene expression of *fas*, *srebp-1*, *acc2*, *acox*, *cpt-1* and *atgl* in hepatopancreas. Different letters indicate significant differences between groups (*p* < 0.05).

Figure 5 shows the results of lipid metabolism-related enzyme activities and relative gene expression. Compared with the FM group, the CAP group exhibited significantly (*p* < 0.05) elevated activities of FAS and ACC in crayfish hepatopancreas, while CPT-1 activity was markedly (*p* < 0.05) reduced. Compared with the CAP group, both the CAPSP2 and CAPSP3 groups exhibited significantly (*p* < 0.05) reduced FAS and ACC activity in crayfish hepatopancreas, alongside markedly (*p* < 0.05) elevated CAP-1 activity. Similarly, relative gene expression of lipid synthesis-related genes *fas*, *srebp-1* and *acc2* in the hepatopancreas of CAP-treated crayfish was significantly (*p* < 0.05) higher than in other groups, whereas relative expression of lipolysis and metabolism-related genes *acox*, *cpt-1*, and *atgl* was significantly (*p* < 0.05) lower than in the FM and CAPSP2 groups.

### 3.5. Metabolomics Analysis

Non-targeted metabolomics was applied to analyze the metabolic effects of phosphorus on crayfish feeding high levels of CAP. Given that the CAPSP2 group exhibited growth and physiological parameters that were not only significantly improved compared to the CAP group but also comparable to the FM control group, it was selected as the representative phosphorus-supplemented group for subsequent metabolomic analysis alongside the CAP and FM groups. Figure S1 shows the quality control assessment for the experiment, wherein the proportion of characteristic peaks with a relative standard deviation (RSD) < 30% reached approximately 65%. The total ion current (TIC) analysis of QC samples demonstrated substantial overlap in response intensity and retention time across all chromatographic peaks. Pearson correlation analysis revealed a correlation coefficient exceeding 0.9 between QC samples. These findings collectively demonstrate excellent experimental reproducibility and minimal variation attributable to instrument error during the testing process. Table S7 shows the number of differentially abundant metabolites across the two modes. Specifically, 2372 and 2741 peaks were identified in the ESI+ and ESI− modes, respectively. Following quality control, 450 and 287 peaks were selected for subsequent analysis.

**Figure 6:**
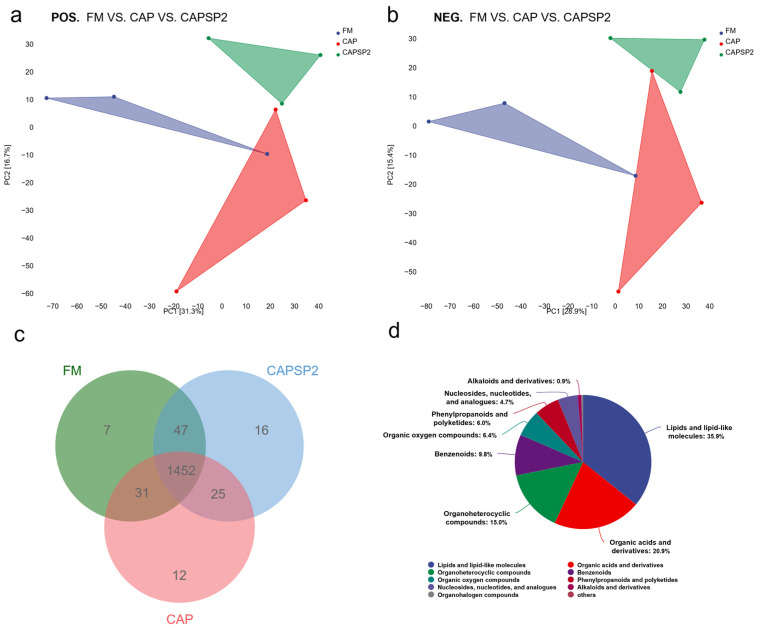
Metabolomic differential analysis of crayfish hemolymph between the FM group, CAP group, and CAPSP2 group. (**a**,**b**) Principal component analysis (PCA) of three groups in POS. and NEG. modes. PC1 represents principal component 1, PC2 represents principal component 2. (**c**) Venn diagrams were used to compare metabolites across groups. The total number of metabolites that were differentially expressed for that comparison group is represented by the sum of the numbers inside the circles. The metabolites that differ in expression between the two comparison groups are shown by the overlapped region. (**d**) The pie chart displays the classification of metabolites into different categories following screening and identification, with different colors representing distinct categories.

Principal Component Analysis (PCA) presented a two-dimensional graphical layout for the three sample groups, effectively illustrating metabolic variations between groups (Figure 6a,b). Outlier detection via OPLS-DA and PLS-DA scatter plots further confirmed distinct metabolic profiles across groups (Figures S2 and S3). Venn diagrams revealed overlapping and unique differentially abundant metabolites: beyond 1452 shared metabolites, the FM, CAP, and CPSP2 groups exhibited 7, 12, and 16 unique metabolites, respectively (Figure 6c). These findings indicate significant metabolic differences in the hepatopancreas of crayfish from the FM, CAP, and CAPSP2 groups, necessitating biomarker screening and analysis of differentially expressed metabolites.

**Figure 7:**
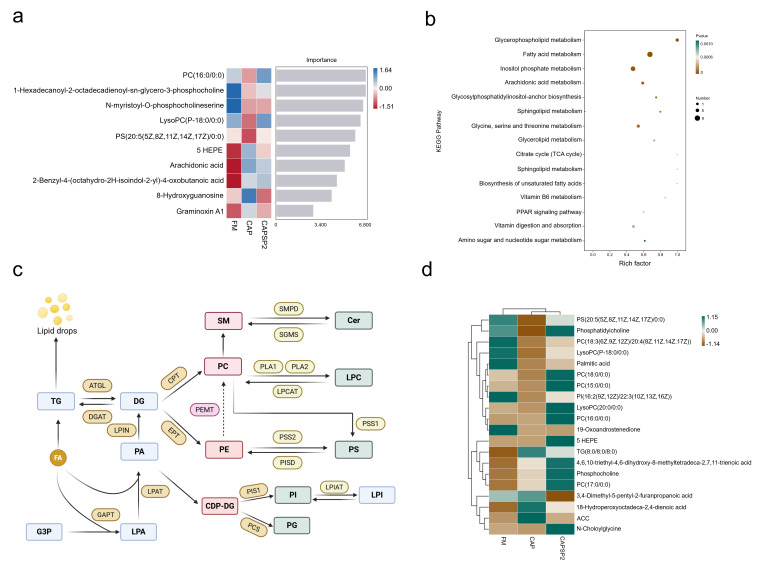
Screening and analysis of key metabolites and pathways. (**a**) Key metabolites predicted by machine learning models constructed using the random forest algorithm. The horizontal axis represents the importance score of species for the classifier model, while the vertical axis shows the names of metabolites. (**b**) KEGG pathway analysis. Based on the KEGG enrichment analysis results for differentially expressed metabolites, the top 15 pathways with the smallest *p*-values (i.e., most significantly enriched) were selected for display: The x-axis represents −log10 (*p* value), and the y-axis represents KEGG Pathway. (**c**) Key Compounds and Pathways in Glycerophospholipid Metabolism. (**d**) Heatmap of relative abundance of metabolites enriched in each group. The relative abundance levels in the figure are represented by different colors, with greener hues indicating higher expression. Columns denote groups, while rows represent metabolite names.

Among all metabolites identified in this project, those constituting over 5% of the total included lipids and lipid-like molecules, organic acids and derivatives, organoheterocyclic compounds, benzenoids, organic oxygen compounds, as well as phenylpropanoids and polyketides. Lipids and lipid-like molecules accounted for the highest proportion at 35.9%, indicating that lipid metabolism in the hepatopancreas of crayfish was significantly affected in this study (Figure 6d). Metabolomics typically employs strict OPLS-DA VIP > 1 and *p* < 0.05 as screening criteria for identifying significantly different metabolites. The key metabolites predicted by the machine learning model constructed via the random forest algorithm primarily comprise glycerophospholipids (GPs) and fatty acyls (FAs) (Figure 7a). The top five contributing metabolites were the various stages of phospholipid metabolism and its intermediates, namely PC (16:0/0:0), 1-Hexadecanoyl-2-octadecadienoyl-sn-glycero-3-phosphocholine, N-Myristoyl-O-phosphocholine serine, LySOPC (P-18:0/0:0), and PS (20:5(5Z,8Z,11Z,14Z,17Z)/10:0). KEGG pathway enrichment analysis further revealed that differentially abundant metabolites predominantly participated in pathways including glycerophospholipid metabolism, fatty acid metabolism, inositol phosphate metabolism, arachidonic acid metabolism, glycosylphosphatidylinositol-anchor biosynthesis, sphingolipid metabolism, and glycine, serine and threonine metabolism (Figure 7b).

The volcano plot showed the overall distribution of differentially expressed metabolites in the hepatopancreas of crayfish from the FM, CAP, and CAPSP2 groups (Figure S4). The relative enrichment abundance of key differential metabolites was presented via a heatmap, primarily involving lipid substances such as Phosphatidylcholine (PC), Phosphatidylserine (PS), and Phosphatidylinositol (PI) within glycerophospholipid metabolism (Figure 7c,d). Among these, the FM and CAPSP2 groups exhibited significantly (*p* < 0.05) higher PC, Lyso-phosphatidylcholine (LPC), PS, and PI contents in crayfish compared to the CAP group, with no significant (*p* > 0.05) difference between the FM and CAPSP2 groups. Furthermore, the CAP group crayfish displayed the highest (*p* < 0.05) contents of TG and ACC (Tables S8–S10).

## 4. Discussion

One of the most significant economic characteristics of aquaculture is the growth of aquatic creatures [41]. In this experiment, the SGR of the CAP group was significantly lower compared to the fishmeal group (containing 10% fishmeal in the basal formulation), indicating that high levels of CAP replacing fishmeal reduced the growth performance of crayfish. This finding was consistent with our previous research [34]. A similar decline in SGR and final body weight was observed when replacing over 45% of fishmeal in turbot (*Scophthalmus maximus* L.) diets (base formulation containing 60% fishmeal) [42]. However, studies on other species such as black sea bream and European seabass (*Dicentrarchus labrax*) found that replacing over 70% of fishmeal with CAP did not impair growth performance, potentially due to species-specific variations in CAP utilization capacity [11,43]. Reduced growth performance has been closely linked to phosphorus deficiencies in diet, according to numerous studies [44,45]. The phosphorus content of the CAP group meal was much lower than the 1.39–1.43% dietary phosphorus requirement for crayfish [35]. This could have caused the crayfish to become phosphorus-deficient, which would have hindered their ability to grow. In this experiment, compared with the CAP group, the SGR of crayfish in the CAPSP2 group and CAPSP3 group was significantly higher than that in the CAP group, indicating that supplementing phosphorus in feed plays a crucial role in improving crayfish growth performance.

The hepatopancreas serves as the primary site for nutrient digestion and absorption and the central hub for lipid metabolism, with its health being crucial for maintaining metabolic and antioxidant balance in the body [46]. Hepatocyte damage leads to the release of ALT/AST into the bloodstream, making them recognized markers of hepatopancreatic injury [47]. This study found that activities of ALT and AST in the hemolymph of CAP-fed crayfish were significantly higher than those in the FM, CAPSP2 and CAPSP3 groups. Concurrently, structural abnormalities observed in the hepatopancreas of CAP-fed crayfish on H&E sections indicated that high-proportion fishmeal replacement with CAP caused hepatopancreatic damage, a phenomenon consistent with findings in largemouth bass [48]. Oxidative stress represents a common pathophysiological mechanism in hepatopancreatic injury progression, with malondialdehyde (MDA) serving as a key biomarker for assessing oxidative damage. In this experiment, elevated malondialdehyde levels and reduced antioxidant enzyme activities and relative gene expression in the CAP group crayfish indicated that high-level CAP replacement of fishmeal led to increased oxidative stress and decreased antioxidant capacity in crayfish, which was supported by the T-AOC results. Similar findings were observed in Pacific white shrimp and pearl gentian grouper, where excessive CAP in feed also reduced antioxidant enzyme activity, disrupting the antioxidant system [7,14]. However, comparing results among the CAPSP2, CAPSP3, and CAP groups revealed that phosphorus supplementation significantly increased antioxidant enzyme activity and relative gene expression levels in crayfish hepatopancreas while reducing MDA content. Phosphorus is an integral component of key molecules in the antioxidant system, such as NADPH (nicotinamide adenine dinucleotide phosphate) [49]. NADPH is essential for regenerating reduced glutathione (GSH) from oxidized glutathione (GSSG) via the enzyme glutathione reductase (GR). Glutathione peroxidase (GPX) utilizes GSH to reduce hydrogen peroxide and lipid peroxides [50]. Furthermore, phosphorus is crucial for ATP synthesis, fueling various cellular processes including the antioxidant response [51]. Therefore, phosphorus deficiency likely compromises the entire antioxidant network by limiting the substrates and energy required for its function. Phosphorus deficiency-induced declines in antioxidant enzyme activity have been documented in both grass carp and blunt snout bream (*Megalobrama amblycephala*) studies [52,53]. Research indicates that oxidative damage induced by low phosphorus suppresses SOD expression via the Keap1/Nrf2 pathway [54]. Furthermore, consistent with the present findings, studies on snakehead (*Channa argus* × *Channa maculata*) and blunt snout bream demonstrated that adding appropriate phosphorus levels to feed reduced the MDA content and enhanced oxidase activity in the fish [55,56]. In summary, these findings indicate that supplementing feed phosphorus levels to 1.40–1.52% alleviated oxidative stress induced by high-dose CAP intake. However, the specific mechanism by which phosphorus enhances antioxidant enzyme activity requires further investigation.

Abnormal lipid deposition is frequently regarded as a key characteristic of lipid metabolism disorders [57]. In this study, the CAP group exhibited the highest TG and TC levels in both hemolymph and hepatopancreas, alongside the Oil Red O staining results, indicating that CAP exacerbated lipid accumulation within crayfish. LDL-C and HDL-C reflected the stability of hepatic-hematological lipid circulation. The significantly elevated LDL-C content in the CAP group crayfish indicated increased lipid transport from the hepatopancreas to the hemolymph, indirectly reflecting exacerbated lipid deposition within the hepatopancreas. Further investigations revealed that high-level fishmeal CAP substitution reduced CPT-1 activity and the relative expression of lipolysis-related genes *acox* and *cpt-1* in crayfish hepatopancreas, while increasing FAS and ACC activity and the relative expression and enzyme activity of lipogenesis-related genes *fas*, *srebp-1*, and *acc2*, which was consistent with findings in tilapia [6]. In lipid metabolism, transcription of nearly all hepatic triglyceride and fatty acid synthesis genes is regulated by SREBP-1 [57]. ACC is a rate-limiting enzyme in lipid synthesis, and FAS overexpression also exacerbates lipid deposition [58]. Furthermore, during lipolysis, CPT-1 and ACOX functions by limiting fatty acid β-oxidation, while ATGL regulates triglyceride hydrolysis [59]. Consequently, elevated CAP levels cause lipid metabolism disorders in crayfish.

However, comparisons between the CAP and CAPSP2 groups revealed that phosphorus supplementation significantly reduced lipid content and the activity and relative gene expression of lipid synthesis-related enzymes in crayfish while simultaneously increasing the activity and relative gene expression of enzymes associated with lipid metabolism. Phosphorus plays a crucial role in lipid metabolism by participating in ATP synthesis and binding lipids to form phospholipids, which aid in fat breakdown and absorption [60]. A study on blunt snout bream indicated that insufficient phosphorus in feed inhibited lipid utilization [52]. Lei et al. [61] observed that low phosphorus caused abnormal lipid deposition in the liver of Chinese mitten crabs (*Eriocheir sinensis*) and significantly upregulated the expression of *srebp-1* and *fas* in the liver, a pattern similar to that seen in the CAP group of crayfish. Excessive lipid deposition under low-phosphorus conditions could stem from phosphorus deficiency inhibiting fatty acid β-oxidation. The conversion of long-chain fatty acids to acyl-CoA requires substantial ATP energy expenditure [62]. Impaired oxidative phosphorylation and insufficient ATP supply due to phosphorus deficiency may inhibit this process, weakening the organism’s ability to oxidatively break down fats and consequently leading to increased fat deposition, while the specific mechanisms require further investigation. However, a study on channel catfish (*Ictalurus punctatus*) indicated that adding appropriate phosphorus levels to feed promoted lipid metabolism and reduced lipid deposition [63]. Therefore, in this study, phosphorus could be a key limiting factor affecting the crayfish’s absorption and utilization of CAP. Appropriate phosphorus supplementation in feed could mitigate lipid metabolism disorders and excessive lipid deposition caused by the high-level CAP replacement of fishmeal.

Nutritional metabolomics in aquatic animals primarily seeks to identify metabolic markers and pathways significantly altered by dietary influences, aiding in understanding the utilization mechanisms of novel feeds under controlled conditions [64]. In this study, non-targeted metabolomics results obtained via LC-MS revealed that significant differences in both the types and levels of metabolites were observed among crayfish in the FM, CAP, and CAPSP2 groups. Metabolic marker and KEGG analyses indicated that the primary altered metabolites and pathways were concentrated in the glycerophospholipid metabolism pathway, suggesting that lipid metabolism disruption in the hepatopancreas of CAP-fed crayfish likely occurs mainly within this pathway. Biomarker results predicted by the random forest algorithm indicated PC and LPC as major differential metabolites, with significantly higher enrichment levels in the CAPSP2 and FM groups compared to the CAP group, which suggest that PC and LPC could be key factors influencing crayfish lipid synthesis metabolism and signal transduction. As major glycerophospholipids, PC and LPC constitute primary components of the bilayer structures in cell membranes and lipoproteins, participating in the biosynthesis of DNA, RNA, and proteins [65]. Pu et al. [66] similarly revealed that crayfish lipid metabolism disorders are closely associated with PC and PA in glycerophospholipid metabolism. Research indicates PC facilitates lipid digestion, absorption, and utilization while reducing serum lipid molecule levels [67]. As a primary constituent and synthetic substrate of phospholipids, phosphorus content is intrinsically linked to the synthesis and metabolism of various glycerophospholipids [68]. Studies in American eels (*Anguilla rostrata*) revealed that phosphorus supplementation significantly upregulated hepatic glycerophospholipid metabolism to enhance fatty acid oxidation [69]. Compared to the CAP group, the CAPSP2 group exhibited markedly increased PC, LPC, and PI contents, indicating that phosphorus supplementation could reverse glycerophospholipid metabolic remodeling induced by high-level CAP intake.

Both glycerophospholipids and glycerophosphocholine demonstrate lipolytic effects by enhancing fatty acid β-oxidation [70]. In turbot, PC significantly suppressed lipid synthesis-related genes (FAS, SREBP-1) while increasing the relative expression of lipolysis-related genes (CPT-1) [71]. Interestingly, our results demonstrated that dietary phosphorus supplementation concurrently elevated the hepatopancreatic levels of PC and LPC and promoted fatty acid β-oxidation (evidenced by suppressed FAS and enhanced CPT-1 activities). This compelling parallel suggests a potential mechanistic link whereby adequate phosphorus availability enhances glycerophospholipid metabolism, leading to increased PC synthesis, which could subsequently facilitate fatty acid catabolism. Thus, it is reasonable to hypothesize that phosphorus could activate glycerophospholipid metabolism to synthesize PC, thereby enhancing fatty acid oxidative breakdown. However, the phosphorus-PC-lipolysis pathway requires further experimental confirmation. In summary, this study observed that high CAP levels remodel metabolic pathways in crayfish. Phosphorus supplementation could alleviate lipid metabolism disorders and abnormal lipid deposition induced by high CAP replacement of fishmeal by enhancing glycerophospholipid metabolism, which further confirms the view that phosphorus is a limiting factor for improving CAP utilization in crayfish.

## 5. Conclusions

This study demonstrated that the complete replacement of fishmeal with Clostridium autoethanogenum protein (CAP) in crayfish diets induced growth retardation, hepatopancreatic damage, oxidative stress, and lipid metabolic dysfunction. These adverse effects were primarily attributable to dietary phosphorus deficiency. Supplementing phosphorus to a level of 1.40–1.52% effectively mitigated these disorders by rectifying glycerophospholipid metabolism, which in turn enhanced hepatic fatty acid oxidation, reduced lipid deposition, and improved antioxidant capacity. Consequently, phosphorus was identified as the critical limiting factor for the efficient utilization of high-dose CAP in *Procambarus clarkii*. To ensure optimal growth and health, the dietary phosphorus level should be maintained at or above 1.40% in crayfish feeds where CAP is used as a primary protein source.

## Figures and Tables

**Table 1:** Formulation and proximate composition of experimental diets.

Ingredients (%, Dry Basis)	FM	CAP	CAPSP1	CAPSP2	CAPSP3
Fish meal	10.00	0.00	0.00	0.00	0.00
Soybean meal	20.26	20.26	20.26	20.26	20.26
Rapeseed meal	14.00	14.00	14.00	14.00	14.00
Cottonseed meal	17.00	17.00	17.00	17.00	17.00
Wheat flour	18.00	18.00	18.00	18.00	18.00
Wheat bran	9.10	9.10	9.10	9.10	9.10
CAP ^1^	0.00	8.09	8.09	8.09	8.09
Fish oil	0.40	1.37	1.37	1.37	1.37
Soy oil	4.00	4.00	4.00	4.00	4.00
α-Starch	2.00	2.00	2.00	2.00	2.00
Ca(H_2_PO_4_)_2_	0.00	0.00	2.50	3.00	3.50
CaCO_3_	1.06	1.78	0.80	0.60	0.40
Microcrystalline cellulose	2.84	3.06	1.54	1.24	0.94
Phagostimulant	0.10	0.10	0.10	0.10	0.10
Premix ^2^	1.00	1.00	1.00	1.00	1.00
Antioxidant	0.01	0.01	0.01	0.01	0.01
Mold inhibitor	0.03	0.03	0.03	0.03	0.03
Choline chloride	0.20	0.20	0.20	0.20	0.20
Proximate composition (%)					
Crude protein	35.02	34.94	34.91	34.86	34.98
Crude lipid	6.53	6.51	6.55	6.61	6.58
Phosphorus	1.41	0.66	1.27	1.40	1.52

^1^ CAP, Clostridium autoethanogenum protein (DM, %), CP 84.00; CL 0.75; phosphorus, 1.40; provided by Qingdao Baiweiyinge Biotechnology Co., Ltd, Qingdao, China. ^2^ Premix was prepared as in our previous work [34].

**Table 2:** Sequences of primer used in real-time PCR.

Gene	Forward (5′ → 3′)	Reverse (5′ → 3′)	Gene Bank No.
*β-actin*	TATCCTGCGTCTGGACTTGG	CGAACGATTTCTCGCTCTGC	KR135165.1
*fas*	AAACTATGGGTGGGCTAACAG	CAGATTTGACGAGCGATGC	MF062033.1
*srebp-1*	GTTTTTCGGCTCTTGGCTGG	CAGGGTTCACCAGGGTTGTT	XM_045746451.1
*acc2*	AGGGTCAGATGTATCGGGTGT	CTTGTGCGAGCAAGAATAAAGT	XM_045732946.1
*acox*	CCAGCGTAACAGCCAGTATG	TATTTCAATGCCCGAGGTAG	XM_045740831.1
*cpt1*	CCTGGGTGTATTGGTCATCG	CAAGGCAAGAGGTAGCATCA	XM_045741250.1
*atgl*	GAAGGGAGTGCCACAAAGTC	CATTCAGCGATGGTCTACGA	XM_045763583.1
*gpx*	CGAACCCTTGATGACCCTG	GAATGTCCCCAATCCTGATG	JN835259.1
*sod*	CTGGCTGCTGCTGGAGTAA	GTGGTGCTTGGAGTGATGAA	KC333178.1

*fas*, fatty acid synthetase; *srebp-1*, sterol regulatory element binding proteins 1; *acc2*, acyl-coA carboxylase 2; *acox*, acyl-coA oxidase 1; *cpt-1*, carnitine palmitoyltransferase 1; *atgl*, adipose triglyceride lipase; *gpx*, glutathione peroxidase; *sod*, superoxide dismutase.

## Data Availability

The original contributions presented in this study are included in the article/Supplementary Material. Further inquiries can be directed to the corresponding author.

## Supplementary Materials

The following supporting information can be downloaded at: https://www.mdpi.com/article/10.3390/antiox15010028/s1, Figure S1: Quality control of metabolomics data. (a) Analysis of relative standard deviation (RSD) in QC. (b) QC correlation analysis. (c) QC mass spectrometry peak pattern comparison. (d) Chromatograms of UPLC analysis, Figure S2: OPLS-DA score plots for pairwise comparisons between groups in both POS and NEG modes, Figure S3: PLS-DA score plots for pairwise comparisons between groups in both POS and NEG modes, Figure S4. Volcano plots for pairwise comparisons between groups in both POS and NEG modes, Table S1: Nutrient composition of fishmeal (FM) and Clostridium autoethanogenum protein (CAP) used in this study (% dry matter), Table S2: Phosphorus content in feed ingredients (%), Table S3: Results of the Shapiro-Wilk test for normality, Table S4: Results of using the Levene test for homogeneity of variance, Table S5: Statistical data simulated using various models, Table S6: Results of MANOVA tests for all variables, Table S7: Information ontotal ion number and identification, Table S8: Differential metabolites in the haemolymph of *P. clarkii *between the FM and CAP groups, Table S9: Differential metabolites in the haemolymph of *P. clarkii *between the CAPSP2 and CAP groups, Table S10: Differential metabolites in the haemolymph of *P. clarkii *between the FM and CAPSP2 groups.

## Abbreviations

The following abbreviations are used in this manuscript:

CAP	*Clostridium autoethanogenum* protein
SGR	Specific growth rate
PC	Phosphatidylcholine
LPC	Lysophosphatidylcholine
T-AOC	Total antioxidant capacity
AST	Aspartate aminotransferase
ALT	Alanine aminotransferase
CAT	Catalase
SOD	Superoxide dismutase
GSH-PX	Glutathione peroxidase
MDA	Malondialdehyde
TG	Triglycerides
TC	Total cholesterol
LDL-C	Low density lipoprotein
HDL-C	High density lipoprotein
*Fas*	Fatty acid synthetase
*srebp-1*	Sterol regulatory element binding proteins 1
*acc2*	Acyl-coA carboxylase 2
*acox*	Acyl-coA oxidase 1
*cpt-1*	Carnitine palmitoyltransferase 1
*atgl*	Adipose triglyceride lipase
