# Supplementary material for: Phosphorus’s Ameliorative Effect on High Level Bacterial Protein-Induced Metabolic Disorders: Alleviating Oxidative Stress and Lipid Dysregulation in Procambarus clarkii
Source: Antioxidants (Basel). 2025 Dec 24;15(1):28. doi: 10.3390/antiox15010028 (PMC12837147; doi:10.3390/antiox15010028)
Supplement: Supplementary file 1 [file antioxidants-15-00028-s001.zip › antioxidants-4037850-supplementary.pdf]

Table S1. Nutrient composition of fishmeal (FM) and Clostridium autoethanogenum protein (CAP) used in this study (% dry matter).

	FM	CAP
Proximate composition		
Crude protein	68.43	84.54
Crude lipid	10.71	0.77
Phosphorus (%)	2.57	1.41
Amino acid composition		
Essential amino acid (EAA)		
Lysine	4.76	7.35
Phenylalanine	3.04	3.14
Leucine	2.92	6.06
Isoleucine	4.85	4.97
Threonine	2 32	4.38
Methionine	1.69	2.32
Valine	3.57	5.06
Delicious amino acid (DAA)		
Alanine	4.06	4.84
Glycine	4.04	3.93
Glutamic acid	9.5	8.55
Aspartic acid	6.17	8.94
Non-essential amino acid (NEAA)		
Cystine	0.47	1.39
Arginine	3.83	3.4
Tyrosine	1.78	2.96
Proline	2.54	2.76
Serine	2.05	3.32
Histidine	1 83	1.21
Amino acid composition		
∑EAA	20.83	33.28
∑DAA	23.77	26.26
∑NEAA	10.67	15.04

Table S2. Phosphorus content in feed ingredients (%).

Ingredients	Phosphorus content (%)
Fish meal	2.50
Soybean meal	0.65
Rapeseed meal	1.02
Cottonseed meal	1.10
Wheat flour	0.41
Wheat bran	0.92
CAP	1.41
Fish oil	0.00
Soy oil	0.00
α-starch	0.00
$Ca(H_2PO_4)_2$	24.60
$CaCO_3$	0.00
Microcrystalline cellulose	0.00
Phagostimulant	0.00
Premix	0.00
Antioxidant	0.00
Mold inhibitor	0.00
Choline chloride	0.00

Table S3. Results of the Shapiro-Wilk test for normality.

	Shapiro-Wilk		
	**Statistic**	**df**	**Sig.**
TC[1]	.953	45	.069
TG[1]	.981	45	.661
LDL-C[1]	.938	45	.078
HDL-C[1]	.961	45	.128
TG[2]	.969	45	.267
TC[2]	.957	45	.104
LDL-C[2]	.988	45	.913
HDL-C[2]	.953	45	.068
ALT[1]	.980	45	.607
AST[1]	.930	45	.073
MDA[2]	.978	45	.554
T-AOC[2]	.976	45	.464
CAT[2]	.920	45	.153
SDD[2]	.980	45	.634
GSH-PX[2]	.958	45	.105
FAS[2]	.931	45	.059
CPT-1[2]	.978	45	.549
ACC[2]	.984	45	.143

[1]Indicator parameters in hemolymph

[2]Indicator parameters in the hepatopancreas

Table S4. Results of using the Levene test for homogeneity of variance.

Levene's Test of Equality of Error Variances[a]

	F	df1	df2	Sig.
TC[1]	1.333	4	40	.274
TG[1]	1.559	4	40	.114
LDL-C[1]	.967	4	40	.436
HDL-C[1]	1.879	4	40	.133
TG[2]	.877	4	40	.486
TC[2]	1.783	4	40	.151
LDL-C[2]	1.632	4	40	.185
HDL-C[2]	1.033	4	40	.208
ALT[1]	.617	4	40	.653
AST[1]	1.816	4	40	.145
MDA[2]	.402	4	40	.806
T-AOC[2]	0.714	4	40	.074
CAT[2]	1.138	4	40	.385
SDD[2]	.086	4	40	.986
GSH-PX[2]	1.411	4	40	.248
FAS[2]	1.405	4	40	.317
CPT-1[2]	1.124	4	40	.359
ACC[2]	0.646	4	40	.213

Tests the null hypothesis that the error variance of the dependent variable is equal across groups.

a. Design: Intercept + groups.

[1]Indicator parameters in hemolymph

[2]Indicator parameters in the hepatopancreas

Table S5. Statistical data simulated using various models.

	Groups					Pr＞F[b]		
	**FM**	**CAP**	**CAPSP1**	**CAPSP2**	**CAPSP3**	**ANOVA**	**Linear**	**Quadratic**
SGR[1] (% /d)	1.93±0.09^{bc}	1.58±0.10^{a}	1.66±0.08^{a}	2.01±0.13^{c}	1.88±0.08^{b}	0.016	0.007	0.096
**Hemolymph**								
TC (mmol/L)	1.66±0.12^{a}	1.98±0.22^{c}	1.85±0.21^{b}	1.71±0.15^{ab}	1.82±0.07^{b}	0.000	0.000	0.002
TG (mmol/L)	0.87±0.03^{a}	0.92±0.04^{b}	0.92±0.01^{b}	0.89±0.02^{a}	0.89±0.03^{a}	0.005	0.053	0.014
HDL-C (mmol/L)	0.36±0.03^{bc}	0.32±0.02^{a}	0.36±0.01^{ab}	0.39±0.02^{c}	0.37±0.01^{c}	0.000	0.153	0.000
LDL-C (mmol/L)	1.92±0.07^{a}	2.49±0.19^{d}	2.29±0.08^{c}	2.01±0.06^{ab}	2.09±0.15^{b}	0.000	0.000	0.000
ALT (U/L)	20.65±3.06^{a}	28.17±2.08^{c}	25.70±1.52^{bc}	22.85±1.71^{ab}	23.20±2.49^{ab}	0.006	0.065	0.006
AST (U/L)	8.75±0.77^{a}	12.43±1.69^{d}	10.40±0.54^{c}	9.63±1.39^{b}	9.32±0.81^{ab}	0.002	0.000	0.000
**hepatopancreas**								
**TC (mmol/g prot)**	0.07±0.01^{a}	0.12±0.01^{c}	0.08±0.02^{a}	0.07±0.01^{a}	0.10±0.01^{b}	0.000	0.212	0.071
**TG (mmol/g prot)**	0.27±0.03^{a}	0.35±0.02^{c}	0.31±0.03^{b}	0.26±0.02^{a}	0.29±0.04^{ab}	0.000	0.000	0.002
**HDL-C (mmol/g prot)**	0.36±0.01^{b}	0.32±0.01^{a}	0.36±0.03^{b}	0.39±0.01^{c}	0.37±0.03^{b}	0.007	0.001	0.000

**LDL-C (mmol/g prot)**	0.10±0.01	0.11±0.0.1	0.09±0.01	0.09±0.01	0.11±0.01	0.084	0.641	0.145
**MDA (nmol/mg prot)**	4.62±0.44[a]	6.07±0.29[c]	5.47±0.33[b]	4.50±0.47[a]	4.79±0.56[a]	0.000	0.000	0.000
**T-AOC (U/mg prot)**	0.50±0.07[b]	0.41±0.04[a]	0.48±0.03[b]	0.47±0.02[b]	0.49±0.04[b]	0.030	0.002	0.019
**CAT (U/mg prot)**	7.07±0.38[b]	6.43±0.54[a]	6.86±0.46[ab]	7.18±0.24[b]	7.22±0.27[b]	0.000	0.317	0.000
**SOD (U/mg prot)**	47.16±4.85[c]	26.87±7.32[a]	34.28±3.91[b]	45.12±4.57[c]	41.76±5.83[c]	0.000	0.106	0.043
**GSH-Px (U/mg prot)**	58.66±3.69[b]	49.35±6.43[a]	56.56±2.74[b]	62.84±5.90[b]	66.56±4.71[b]	0.010	0.006	0.112
**FAS (ng/mL)**	13.04±2.05[b]	18.58±0.73[c]	17.36±1.05[c]	10.10±3.07[a]	11.23±0.99[ab]	0.021	0.009	0.115
**CPT-1 (ng/mL)**	40.72±5.54[bc]	22.12±7.49[a]	35.47±5.46[b]	45.48±8.78[c]	47.69±4.81[c]	0.004	0.003	0.107
**ACC (ng/mL)**	28.27±0.67[a]	36.73±3.48[c]	32.49±2.64[b]	29.13±1.52[a]	30.12±4.73[ab]	0.001	0.000	0.124
Lipid droplet (%)	23.80±5.60[a]	37.20±7.80[b]	34.50±4.30[ab]	27.00±4.20[a]	31.60±6.40[a]	0.004	0.064	0.006
Vecuoles (%)	8.90±2.20[a]	27.40±5.40[b]	14.30±2.40[a]	12.50±4.60[a]	19.40±5.10[ab]	0.013	0.217	0.003

Different letters indicate significant differences between groups ($P < 0.05$).

Table S6. Results of MANOVA tests for all variables.

Source	Dependent Variable	Type III Sum of Squares	df	Mean Square	F	Sig.	Partial Eta Squared
Corrected Model	TC[1]	.601[a]	4	.150	41.066	.000	.804
	TG[1]	.022[b]	4	.006	6.196	.001	.383
	LDL-C[1]	1.900[c]	4	.475	91.950	.000	.902
	HDL-C[1]	43.258[d]	4	10.814	13.825	.000	.580
	TG[2]	.046[e]	4	.012	19.177	.000	.657
	TC[2]	.014[f]	4	.004	49.788	.000	.833
	LDL-C[2]	.001[g]	4	.000	2.232	.083	.182
	HDL-C[2]	.021[h]	4	.005	54.742	.000	.846
	ALT[1]	300.561[i]	4	75.140	14.905	.000	.598
	AST[1]	73.161[j]	4	18.290	58.557	.000	.854
	MDA[2]	15.886[k]	4	3.971	21.343	.000	.681
	T-AOC[2]	.040[l]	4	.010	5.439	.001	.352
	CAT[2]	3.795[m]	4	.949	6.100	.001	.379
	SDD[2]	2529.272[n]	4	632.318	28.363	.000	.739
	GSH-PX[2]	1538.280[o]	4	384.570	19.435	.000	.660
	FAS[2]	504.392[p]	4	126.098	39.330	.000	.797
	CPT-1[2]	3737.398[q]	4	934.349	25.052	.000	.715
	ACC[2]	416.046[r]	4	104.012	18.181	.000	.645
Intercept	TC[1]	146.988	1	146.988	40206.492	.000	.999
	TG[1]	36.506	1	36.506	40350.680	.000	.999

	LDL-C[1]	210.592	1	210.592	40763.072	.000	.999
	HDL-C[1]	2951.839	1	2951.839	3773.600	.000	.990
	TG[2]	3.947	1	3.947	6581.091	.000	.994
	TC[2]	.365	1	.365	5042.468	.000	.992
	LDL-C[2]	.519	1	.519	5668.331	.000	.993
	HDL-C[2]	5.932	1	5.932	61132.842	.000	.999
	ALT[1]	26161.284	1	26161.284	5189.536	.000	.992
	AST[1]	4598.430	1	4598.430	14721.963	.000	.997
	MDA[2]	1167.018	1	1167.018	6271.491	.000	.994
	T-AOC[2]	9.986	1	9.986	5373.960	.000	.993
	CAT[2]	2176.578	1	2176.578	13995.308	.000	.997
	SDD[2]	68568.639	1	68568.639	3075.634	.000	.987
	GSH-PX[2]	155565.702	1	155565.702	7862.007	.000	.995
	FAS[2]	8897.070	1	8897.070	2774.983	.000	.986
	CPT-1[2]	65990.986	1	65990.986	1769.377	.000	.978
	ACC[2]	44222.323	1	44222.323	7730.010	.000	.995
groups	TC[1]	.601	4	.150	41.066	.000	.804
	TG[1]	.022	4	.006	6.196	.001	.383
	LDL-C[1]	1.900	4	.475	91.950	.000	.902
	HDL-C[1]	43.258	4	10.814	13.825	.000	.580
	TG[2]	.046	4	.012	19.177	.000	.657
	TC[2]	.014	4	.004	49.788	.000	.833
	LDL-C[2]	.001	4	.000	2.232	.083	.182
	HDL-C[2]	.021	4	.005	54.742	.000	.846
	ALT[1]	300.561	4	75.140	14.905	.000	.598

	AST[1]	73.161	4	18.290	58.557	.000	.854
	MDA[2]	15.886	4	3.971	21.343	.000	.681
	T-AOC[2]	.040	4	.010	5.439	.001	.352
	CAT[2]	3.795	4	.949	6.100	.001	.379
	SDD[2]	2529.272	4	632.318	28.363	.000	.739
	GSH-PX[2]	1538.280	4	384.570	19.435	.000	.660
	FAS[2]	504.392	4	126.098	39.330	.000	.797
	CPT-1[2]	3737.398	4	934.349	25.052	.000	.715
	ACC[2]	416.046	4	104.012	18.181	.000	.645
Error	TC[1]	.146	40	.004			
	TG[1]	.036	40	.001			
	LDL-C[1]	.207	40	.005			
	HDL-C[1]	31.289	40	.782			
	TG[2]	.024	40	.001			
	TC[2]	.003	40	7.231E-5			
	LDL-C[2]	.004	40	9.153E-5			
	HDL-C[2]	.004	40	9.704E-5			
	ALT[1]	201.646	40	5.041			
	AST[1]	12.494	40	.312			
	MDA[2]	7.443	40	.186			
	T-AOC[2]	.074	40	.002			
	CAT[2]	6.221	40	.156			
	SDD[2]	891.766	40	22.294			
	GSH-PX[2]	791.481	40	19.787			
	FAS[2]	128.247	40	3.206			

	CPT-1[2]	1491.847	40	37.296	
	ACC[2]	228.834	40	5.721	
Total	TC[1]	147.734	45		
	TG[1]	36.565	45		
	LDL-C[1]	212.699	45		
	HDL-C[1]	3026.386	45		
	TG[2]	4.017	45		
	TC[2]	.382	45		
	LDL-C[2]	.523	45		
	HDL-C[2]	5.957	45		
	ALT[1]	26663.491	45		
	AST[1]	4684.085	45		
	MDA[2]	1190.347	45		
	T-AOC[2]	10.100	45		
	CAT[2]	2186.594	45		
	SDD[2]	71989.677	45		
	GSH-PX[2]	157895.464	45		
	FAS[2]	9529.709	45		
	CPT-1[2]	71220.230	45		
	ACC[2]	44867.203	45		
Corrected Total	TC[1]	.747	44		
	TG[1]	.059	44		
	LDL-C[1]	2.107	44		
	HDL-C[1]	74.547	44		
	TG[2]	.070	44		

TC[2]	.017	44
LDL-C[2]	.004	44
HDL-C[2]	.025	44
ALT[1]	502.208	44
AST[1]	85.655	44
MDA[2]	23.329	44
T-AOC[2]	.115	44
CAT[2]	10.016	44
SDD[2]	3421.038	44
GSH-PX[2]	2329.761	44
FAS[2]	632.639	44
CPT-1[2]	5229.245	44
ACC[2]	644.881	44

[1]Indicator parameters in hemolymph

[2]Indicator parameters in the hepatopancreas

R Squared = .383 (Adjusted R Squared = .321)[b]

R Squared = .902 (Adjusted R Squared = .892)[c]

R Squared = .580 (Adjusted R Squared = .538)[d]

R Squared = .657 (Adjusted R Squared = .623)[e]

R Squared = .833 (Adjusted R Squared = .816)[f]

R Squared = .182 (Adjusted R Squared = .101)[g]

R Squared = .846 (Adjusted R Squared = .830)[h]

R Squared = .598 (Adjusted R Squared = .558)[i]

R Squared = .854 (Adjusted R Squared = .840)[j]

R Squared = .681 (Adjusted R Squared = .649)[k]

R Squared = .352 (Adjusted R Squared = .288)[l]

R Squared = .379 (Adjusted R Squared = .317)[m]

R Squared = .739 (Adjusted R Squared = .713)[n]

R Squared = .660 (Adjusted R Squared = .626)[o]

R Squared = .797 (Adjusted R Squared = .777)[p]

R Squared = .715 (Adjusted R Squared = .686)[q]

R Squared = .645 (Adjusted R Squared = .610)[r]

Table S7. Information ontotal ion number and identification.

mode	Total ion number	Rsd < 30% ion number	Metabolites in library	Metabolites in kegg
ESI+	2372	450	230	137
ESI-	2741	287	172	86

Table S8. Differential metabolites in the haemolymph of *P. clarkii* between the FM and CAP groups.

Differential metabolites	VIP [a]	*P* value [b]	FC [c]	Levels [d]
Phosphatidyicholine	5.14	2.54×10^{-2}	1.53	Up
PC(18:0/0:0)	3.94	3.43×10^{-5}	4.71	Up
Hydantoin-5-propionic acid	8.88	6.55×10^{-4}	2.67	Up
PS(20:5(5Z,8Z,11Z,14Z,17Z)/0:0)	2.03	1.26×10^{-3}	2.56	Up
8-Isoprostaglandin E2	1.09	8.26×10^{-7}	4.87	Up
Phosphocholine	5.43	2.71×10^{-4}	2.05	Up
1-Palmitoylphosphatidylcholine	8.65	3.49×10^{-4}	6.12	Up
PC(15:0/0:0)	1.12	9.72×10^{-3}	3.82	Up
LysoPC(P-18:0/0:0)	3.49	4.12×10^{-3}	0.37	Down
Gibberellin A52	4.87	1.29×10^{-4}	0.16	Down
Docosahexaenoic Acid	1.24	2.94×10^{-2}	0.52	Down
8-Iso prostaglandin A2	1.02	8.95×10^{-5}	0.21	Down
3-Oxotetradecanoic acid	4.67	3.26×10^{-4}	0.41	Down

[a] Variable importance in the projection (VIP) was acquired from the OPLS-DA model with a threshold of 1.0.

[b] *P* values were calculated from a two-tailed Student's t-test.

[c] FC = fold change, mean value of peak area obtained from the FM group /mean value of peak area obtained from the CAP group. If the FC value is > 1, it means that metabolites in the FM are more than in the CAP.

[d] Up: an increase in the FM group, down: a decrease in the FM group.

Table S9. Differential metabolites in the haemolymph of *P. clarkii* between the CAPSP2 and CAP groups.

Differential metabolites	VIP [a]	*P* value [b]	FC [c]	Levels [d]
PE(20:3(8Z,11Z,14Z)/22:6(4Z,7Z,10Z,13Z,16Z,19Z))	1.77	4.38×10^{-5}	4.65	Up
5-Acetoxydihydrotheaespirane	2.41	5.02×10^{-3}	5.59	Up
Estradiol cypionate	1.85	3.79×10^{-4}	3.48	Up
PC(18:1(11Z)/18:1(11Z))	2.07	1.16×10^{-3}	9.14	Up
LysoPC(20:0/0:0)	4.87	2.03×10^{-2}	6.39	Up
PC(18:3(6Z.9Z.12Z)/20:4(8Z.11Z.14Z.17Z))	2.03	3.48×10^{-4}	3.30	Up
Flupentixol	1.60	2.21×10^{-3}	7.81	Up
PC(15:0/0:0)	1.76	1.69×10^{-6}	5.51	Up
Palmitoleic acid	3.03	2.62×10^{-3}	1.95	Up
Phosphocholine	2.83	5.52×10^{-5}	3.30	Up
4-Ethylphenol	1.21	3.70×10^{-4}	1.41	Up
LysoPE(18:2(9Z,12Z)/0:0)	3.46	1.93×10^{-6}	2.71	Up
2'-O-Methyluridine	3.63	3.57×10^{-5}	3.43	Up
PC(18:3(6Z.9Z.12Z)/20:4(8Z.11Z.14Z.17Z))	2.44	1.52×10^{-2}	4.26	Up
ILICICOLIN B	1.93	8.23×10^{-3}	5.18	Up
3,4-Dimethyl-5-pentyl-2-furanpropanoic acid	2.29	5.24×10^{-4}	3.57	Up
Calystegine A6	3.01	4.27×10^{-3}	1.95	Up
Phosphatidyicholine	1.53	2.92×10^{-3}	3.34	Up
Naringenin	6.35	1.75×10^{-7}	2.57	Up
MFCD00004183	1.99	5.52×10^{-4}	3.11	Up
Serylisoleucine	3.63	2.16×10^{-7}	0.37	Down
Melagatran	3.99	2.23×10^{-5}	0.21	Down
6''-O-Malonylglycitin	4.12	4.97×10^{-4}	0.27	Down
Valylasparagine	1.57	3.14×10^{-3}	0.34	Down

[a] Variable importance in the projection (VIP) was acquired from the OPLS-DA model with a threshold of 1.0.

[b] *P* values were calculated from a two-tailed Student's t-test.

[c] FC = fold change, mean value of peak area obtained from the CAPSP2 group /mean value of peak area obtained from the CAP group. If the FC value is > 1, it means that metabolites in the CAPSP2 are more than in the CAP.

[d] Up: an increase in the CAPSP2 group, down: a decrease in the CAPSP2 group.

Table S10. Differential metabolites in the haemolymph of *P. clarkii* between the FM and CAPSP2 groups.

Differential metabolites	VIP [a]	*P* value [b]	FC [c]	Levels [d]
8-Isoprostaglandin E2	1.94	3.70×10^{-4}	1.34	Up
18-Hydroperoxyoctadeca-2,4-dienoic acid	2.41	1.81×10^{-2}	1.78	Up
3,4-Dimethyl-5-pentyl-2-furanpropanoic acid	1.39	5.51×10^{-3}	1.12	Up
Graminoxin A1	2.55	2.77×10^{-3}	1.49	Up
5 HEPE	3.17	3.45×10^{-2}	1.35	Up
Astaxanthin	1.26	4.02×10^{-4}	1.46	Up
PS(20:5(5Z,8Z,11Z,14Z,17Z)/0:0)	1.62	5.25×10^{-3}	0.64	Down

[a] Variable importance in the projection (VIP) was acquired fro5m the OPLS-DA model with a threshold of 1.0.

[b] *P* values were calculated from a two-tailed Student's t-test.

[c] FC = fold change, mean value of peak area obtained from the FM group /mean value of peak area obtained from the CAPSP2 group. If the FC value is > 1, it means that metabolites in the FM are more than in the CAPSP2.

[d] Up: an increase in the FM group, down: a decrease in the FM group.

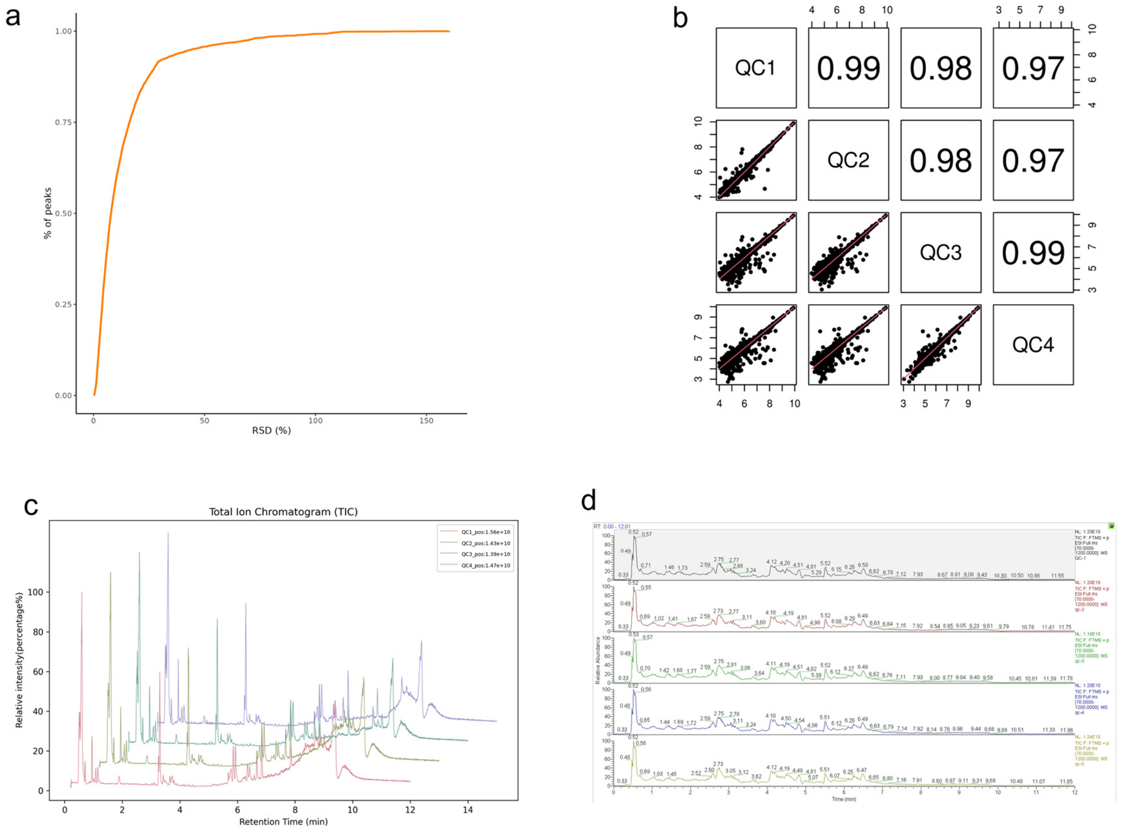

Figure S1. Quality control of metabolomics data. (a) Analysis of relative standard deviation (RSD) in QC. (b) QC correlation analysis. (c) QC mass spectrometry peak pattern comparison. (d) Chromatograms of UPLC analysis.

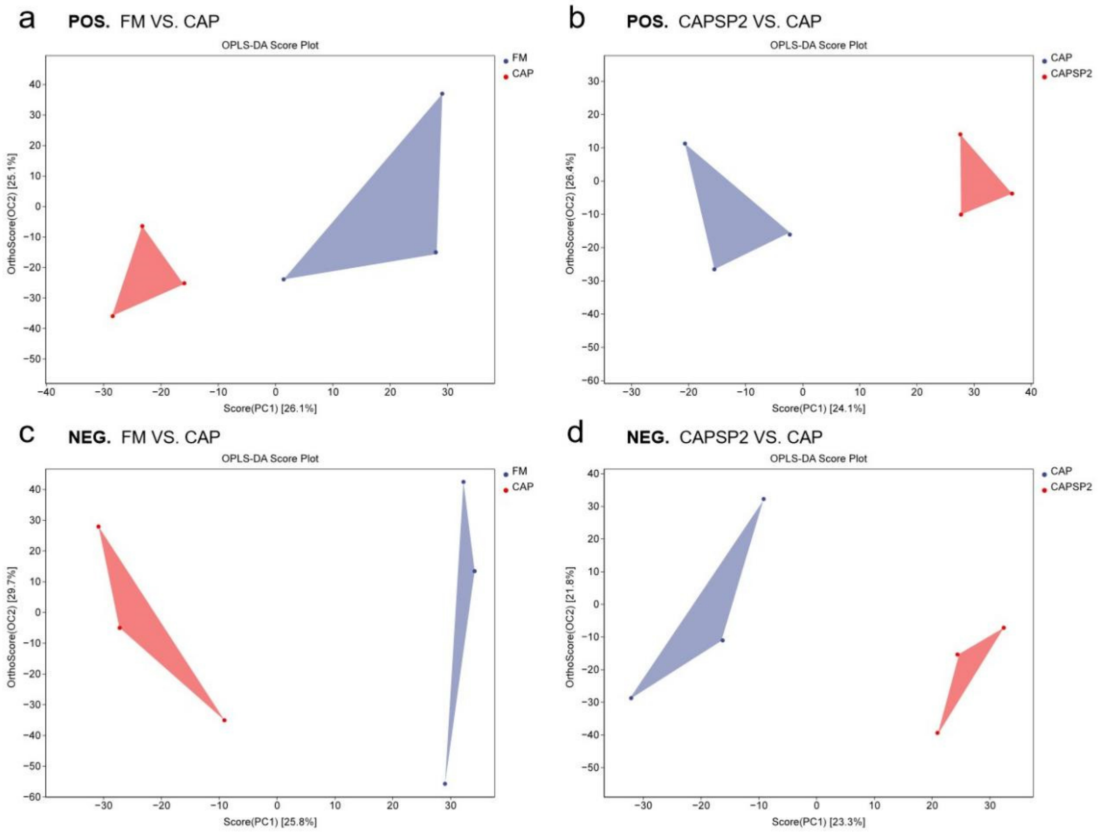

Figure S2. OPLS-DA score plots for pairwise comparisons between groups in both POS and NEG modes.

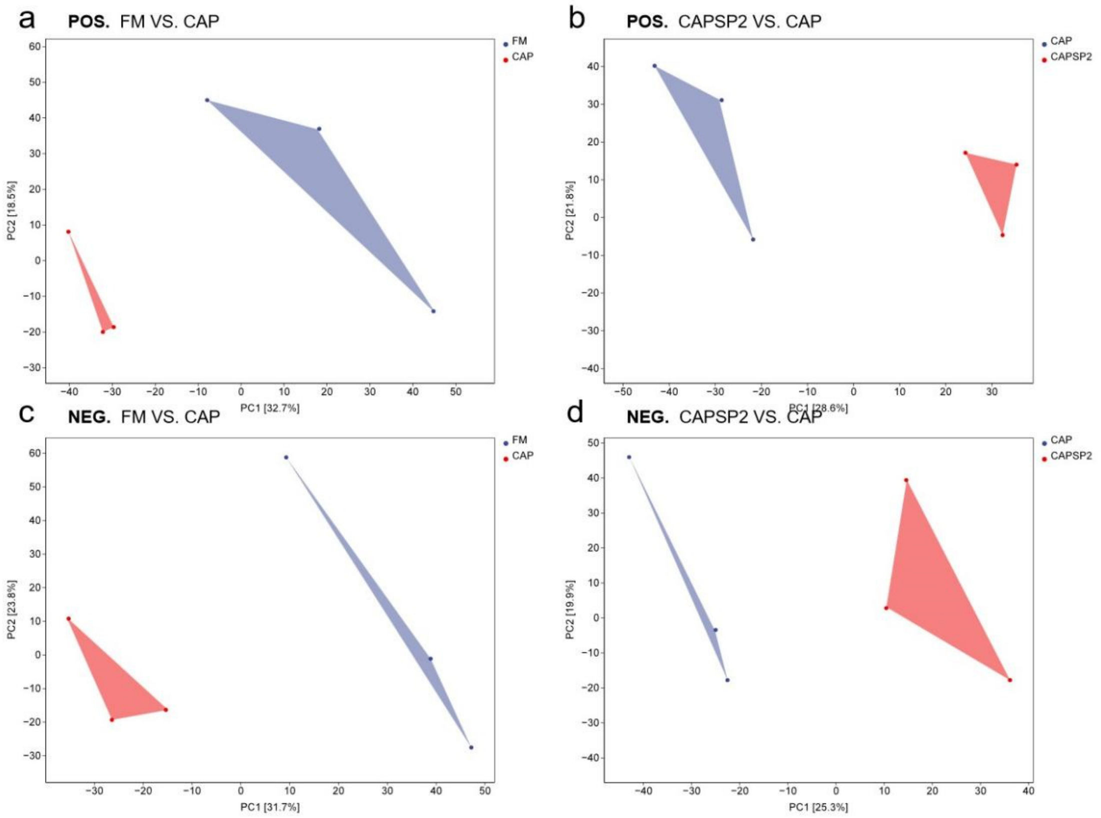

Figure S3. PLS-DA score plots for pairwise comparisons between groups in both POS and NEG modes.

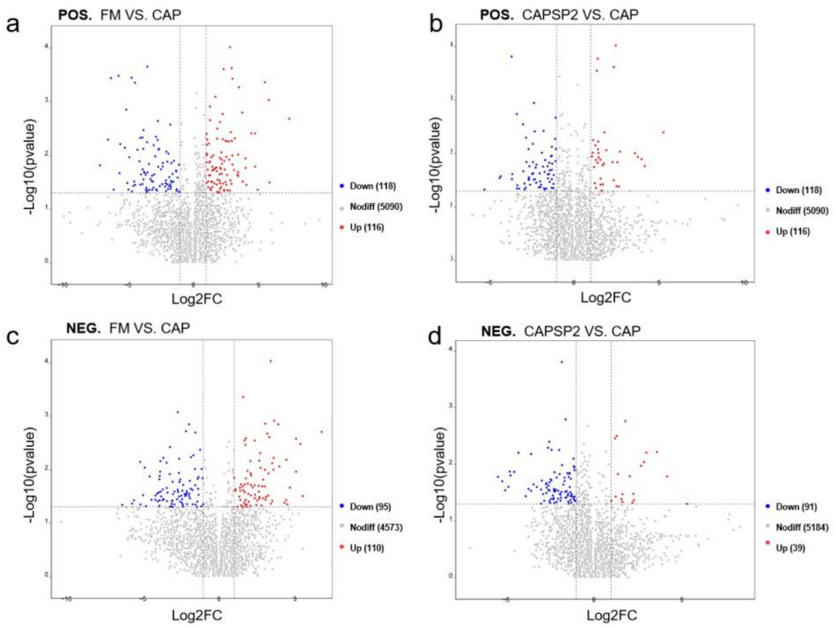

Figure S4. Volcano plots for pairwise comparisons between groups in both POS and NEG modes.
